# Community engagement in maternal and perinatal death surveillance and response (MPDSR): Realist review protocol

**DOI:** 10.12688/wellcomeopenres.18844.1

**Published:** 2023-03-15

**Authors:** Mary Mbuo, Immaculate Okello, Loveday Penn-Kekana, Merlin Willcox, Anayda Portela, Francesca Palestra, Matthews Mathai

**Affiliations:** 1 Public health, Environments and Society, London School of Hygiene & Tropical Medicine, London, WC1E 7HT, UK; 2 Primary Care Research Centre , Aldermoor Health Centre, University of Southampton, Southhampton, UK; 3 Maternal, Newborn, Child and Adolescent Health and Ageing, World Health Organization, Geneva, Switzerland

**Keywords:** Maternal and Perinatal Death Surveillance and Response (MPDSR), Community engagement, Realist Review, Protocol

## Abstract

**Background: **While there has been a decline in maternal and perinatal mortality, deaths remain high in sub-Saharan Africa and Asia. With the sustainable development goals (SDGs) targets to reduce maternal and perinatal mortality, more needs to be done to accelerate progress and improve survival. Maternal and perinatal death surveillance and response (MPDSR) is a strategy to identify the clinical and social circumstances that contribute to maternal and perinatal deaths. Through MPDSR, an active surveillance and response cycle is established by bringing together different stakeholders to review and address these social and clinical factors.

Community engagement in MPDSR provides a strong basis for collective action to address social factors and quality of care issues that contribute to maternal and perinatal deaths. Studies have shown that community members can support identification and reporting of maternal and/or perinatal deaths. Skilled care at birth has been increasing globally, but there are still gaps in quality of care. Through MPDSR, community members can collaborate with health workers to improve quality of care. But we do not know how community engagement in MPDSR works in practice; for whom it works and what aspects work (or do not work) and why. This realist review answers the question: which strategies of community engagement in MPDSR produce which outcomes in which contexts?

**Methods** **: **For this realist review, we will identify published and grey literature by searching relevant databases for articles. We will include papers published from 2004 in all languages and from all countries.

We have set up an advisory group drawn from academia, international organizations, and practitioners of both MPDSR and community engagement to guide the process.

**Conclusion: **This protocol and the subsequent realist review will use theoretical approaches from the community engagement literature to generate theory on community engagement in MPDSR.

**Prospero registration number**:  CRD42022345216

## Introduction

Understanding exactly why a woman and/or her newborn died in pregnancy, around the time of childbirth or in the postnatal period is a crucial first step towards preventing other women and new-borns dying in the same way ^1^. In addition to identifying the medical causes of death, it is important to understand the woman or baby’s personal story and the precise circumstances of the maternal or perinatal death ^2^.

Maternal and Perinatal Death Surveillance and Response (MPDSR) involves qualitative, in-depth review of the causes and circumstances surrounding maternal and perinatal deaths ^3^. Through an active surveillance process, all maternal and perinatal deaths within health facilities and in communities should be identified and reported ^3, 4^. This is followed by a review and response process which involves making recommendations and implementing them. MPDSR can be an integral part of quality of care improvement efforts by addressing the modifiable factors that contributed to a maternal or perinatal death ^4, 5^. MPDSR also involves monitoring the implementation of recommendations made throughout the action cycle and establishing accountability by linking data to actionable solutions ^4^.

The MPDSR process has evolved over time beginning with the WHO -Beyond the Numbers (BTN) in 2004. In 2013, WHO and partners developed the technical guidelines for maternal death surveillance and response (MDSR) ^2^. MDSR links surveillance data to response and improve accountability of the MDSR process. Following the launch of the Every Newborn Action Plan ^6^ and Making Every Baby Count guidelines ^7^, perinatal death surveillance and response was added to the MDSR process in 2017 to leverage on the gains made through BTN and MDSR ^3, 7^. MPDSR can implemented using different tools or strategies in different contexts. These include maternal and/or perinatal death reviews, community based reviews and confidential enquiries into maternal deaths ^8^.

While there has been a decline in maternal and perinatal mortality and an increase in the number of women who deliver in health facilities ^9, 10^, high rates of maternal and perinatal mortality persist in parts of Asia and sub-Saharan Africa ^11– 13^. The overall magnitude of mortality trends remains unclear because of weak surveillance systems especially at the community ^14^. With the sustainable development goal (SDG) target to reduce the global maternal mortality ratio to <70/100000 livebirths, and neonatal mortality rate of 12/1000 live births for every country, more needs to be done to accelerate progress and improve survival rates ^10, 15^. To achieve these global targets, there is need for broad stakeholder participation in understanding, when, where and why maternal and perinatal deaths are happening.

The MPDSR policy and strategies that support its implementation require broad stakeholder participation for effective MPDSR implementation ^2^. Community members are a key stakeholder to the MPDSR processes because they can provide the necessary information that is critical to exploring the social factors and quality of care issues that contribute to the deaths ^3^. Community members can also be involved in advocacy with health workers and policy makers to ensure that the identified recommendations are implemented ^16^.

### Community engagement

Community engagement is a process of developing relationships that enable community members and health professionals to work together for purposes of improving health care ^17^. Community engagement is a complex social process ^18^ with varying terms that are often used interchangeably to describe it; such as community involvement ^19^, community mobilization ^20^, community collaboration ^21^, community participation ^22^ and health co-production ^23^. Central to all the different terms used to describe community engagement are the concepts of (i) the actors involved i.e. community members or community groups and health professionals, (ii) the relationships between the actors and how issues such as social and power hierarchies among the participants affect the engagement process, (iii) recognition and value of the capacities and assets that both health professionals and community members bring to the engagement process (iv) capacity building to address the gaps in skills and experience that both health professionals and community members lack, and (v) the purposes or rationale for engaging community members in the intervention for instance to improve health seeking behaviour ^18, 23– 25^.

Community engagement in MPDSR is anchored in global policies and guidelines. There are several global policies and guidelines that recognize the role that community members can play in the implementation of MPDSR. These include WHO technical guidelines on maternal death surveillance and response (MDSR) ^2^, which identifies community members as a critical stakeholder in surveillance because they can provide information on the social factors that contribute to maternal deaths. The Global Strategy for Women’s Children’s and Adolescent Health ^26^, the Ending preventable maternal mortality initiative ^27^ and the Commission on Information and Accountability for Women’s and Children’s Health ^28^ identify community engagement in MDSR as a necessary component for improving data collection on maternal deaths and empowering communities to engage in social accountability for maternal/perinatal mortality prevention. WHO has also published materials to support the implementation of MPDSR, which include some guiding principles on community engagement ^3^.

Community members could play a role in improving quality of care by providing feedback to health workers on their experiences of receiving healthcare ^4^. A UK study on the quality of perinatal death reviews, recommended that inclusion of parents and parent advocates could improve the review process by creating opportunities for feedback between health professionals and parents/parent advocates ^29^. Secondly, community engagement in MPDSR could help explain why despite an increase in the number of women giving birth in health facilities, studies also show that effective coverage has not increased and high mortality within health facilities persists ^13, 30^.

Community members can also provide information on the social circumstances in which the pregnant woman lived and the circumstances of her death, which can be powerful narratives that provide valuable information for the review and response process ^31, 32^. While there are studies that have demonstrated that community engagement is an important component for the MPDSR process, they have not shown how community engagement in MPDSR works in practice; for whom community engagement works and what aspects of community engagement in MPDSR work (or don’t work) and why.

We propose a realist review approach ^33^ to explore and explain what, why, how and for whom community engagement in MPDSR works (or does not work) to support implementation of MPDSR throughout the action cycle.

### What do we mean by community in the context of MPDSR?

The concept of community is not always well defined in the literature on community participation in health ^24, 34^. In defining who constitutes the community in MPDSR, we have borrowed from the general literature on community participation in health and applied it to the MPDSR context.

See Box 1 for a summary of what we mean by community in the context of MPDSR.

**Box 1: Who is the community in the MPDSR context?**

**People with shared geography and social systems:** the use of the term community participation in the literature tends to focus on people living in the same geographical areas ^21^. It is often expected that people with shared geography share some social systems such as language, values and practices though this is not always the case ^35^. The literature on MPDSR often describes community members on the basis of geographical location with people living in the same area described as community members for instance when conducting verbal and social autopsy ^16, 36, 37^.

**Bereaved family and relatives**: The idea of community can also be used to describe a group of people who have a shared experience ^38^. In the context of MPDSR, some studies have included parents in perinatal death reviews ^29^. Other studies have included relatives of deceased persons as key informants for verbal and social autopsy ^32, 39, 40^ as well as participants in facility review processes ^16^.

**Community representatives**: are people who are appointed or selected to participate on behalf of other community members ^35, 41^. The selection/appointment process is often based on some established criteria such as level of education, level of community influence or social networks in the community ^42^. Community representatives can include village elders, members of health facility committees, community health volunteers/workers, who may be paid or unpaid ^35, 41, 42^. This group of community representatives participate in MPDSR on behalf of the community ^16, 39, 43, 44^.

**Civil society groups/grassroots organization**: these are non-state and not for profit actors including community-based organizations that are formally organized ^45^. The literature on community participation in MPDSR has shown that CSOs and other non-state actors can participate in MPDSR processes, primarily to support community advocacy efforts ^3, 30, 46, 47^.

### Why a realist review

Realist reviews are suitable for providing explanations on how complex interventions work, for whom and under what circumstances they work ^33^. Interventions are described as complex if they have several components to them, or work at different levels of a system or if the different components of the system also interact or are influenced by the external environment ^48^. 

MPDSR has been described as a complex intervention that is implemented at various levels of the health system: national, sub-regional, regional and within health facilities and in the community ^2, 49^. Similarly, community engagement has been described as a complex process that involves several distinct but inter-related concepts such as capacity of the actors, hierarchies between health professionals and the community and social cultural dynamics that govern social interaction ^18, 50^. Given the complexities of both the intervention i.e., MPDSR and community engagement as a process, a realist review is best suited to explain the relationships between the contexts, mechanisms, and outcomes for community engagement in MPDSR.

Realist synthesis is a theory-driven approach that begins with programme theories to describe the underlying assumptions of how an intervention works, the contexts in which it works and for whom it works and the outcomes that result from that interaction ^33^. Realist reviews are increasingly being used to study complex, heterogeneous health-interventions to generate midrange theories on how interventions work ^51, 52^. Realist reviews provide explanations on why interventions work (or don’t work) thus providing pathways to better understand how outcomes are produced in different contexts ^52, 53^. Realist reviews use inductive and abductive reasoning to explore relationships between the outcomes and the contexts and mechanisms i.e. CMO (context-mechanism-outcome) configurations ^51^. The CMO is the basic unit of data that is interpreted to either confirm, refute or refine initial programme theories ^33, 51^

Context can be broadly understood as any condition that triggers and/or modifies the mechanism ^54^. In the context of community engagement in MPDSR, contexts can include (but not limited to): (i) the level of the health system that community engagement in MPDSR is implemented; whether MPDSR is implemented at national, regional, district or health facility, (ii) the policy context e.g. legal frameworks in which MPDSR is implemented, or (iii) the institutional arrangements that support implementation MPDSR such as governments or non-governmental organizations.

A mechanism is the generative force that leads to outcomes and often denotes the resources and the reasoning that lead to either a positive or negative outcome ^52, 55^. For this review, examples of mechanisms could be that in contexts where community members can sanction health professionals and take legal action because there is no legal framework on blame culture in MPDSR, health professionals may be unwilling to allow community members to participate in MPDSR processes due to fear of litigation. In this case, fear of litigation is the mechanism that produces a negative outcome.

Outcomes can be either intended or unintended and can be positive or negative based on how mechanisms and context interact ^54^. For example, the outcome of an MPDSR process in contexts where community members can sanction health professionals can be the exclusion of community members from MPDSR processes by health providers.

### Aim of realist review

The research question is: **which strategies of community engagement in MPDSR produce which outcomes in which contexts?** In synthesizing the evidence on community engagement in MPDSR, to respond to the research question, we will break down the main research question into component parts for clarity as follows:

1. Which activities do community members engage in during MPDSR implementation? We will look at the various parts of the MPDSR cycle and describe how community members are involved.
2. Which contexts influence different mechanisms to produce positive or negative outcomes for community engagement in MPDSR?
3. What are the mechanisms for engaging community members in MPDSR?
4. What are the intended and unintended outcomes of community engagement in MPDSR from the perspective of health workers, community members and policy makers?

By answering these questions, we will generate context-mechanism-outcome (CMOs) configurations to explain why, how, to what extent and for whom community engagement in MPDSR works (or doesn’t work)?

## Methods

This protocol sets out the scope for the realist review based on the approach described by Pawson and colleagues ^33^ with updated step by step guidance by Gilmore and colleagues ^56^. We describe the initial programme theories for community engagement in MPDSR to show why, how and for whom community engagement in MPDSR may work. These initial programme theories (IPTs) are hypotheses derived from an initial scoping of the literature and discussions with key informants who are experts in either MPDSR or community engagement. We describe the stages for conducting the realist review below.

### Registration

This realist review is registered on Prospero; registration number: CRD42022345216.

### Stage 1: Conducting initial scoping search

**Figure 1.**
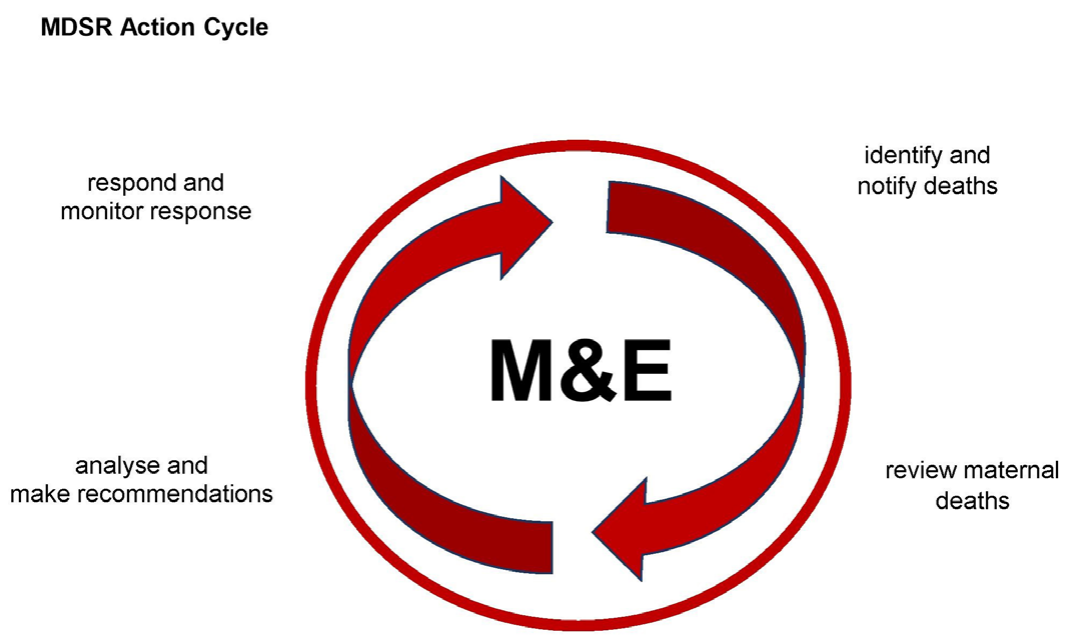
MDSR continuous action cycle. (Reproduced with permission from WHO Maternal Death Surveillance and Response: Technical Guide) ^2^.

MPDSR is implemented through an action cycle and involves surveillance and response. See Figure 1.

There are several steps in the MPDSR cycle; community members can participate in any or all of the following steps during MPDSR implementation.

(i) Identification and notification of maternal and perinatal deaths occurring in the community and providing this information to health workers.
(ii) Review of cases through death review meetings within health facilities or in the community using social autopsy and verbal autopsy. Identifying action points or recommendations to address the modifiable or avoidable factors identified through the review process.
(iii) Implementing the actions or recommendations made during the review process.
(iv) Monitoring and evaluating the implementation of the recommendations. This part of the cycle also involves establishing accountability through advocacy with decision makers and policy actors to ensure implementation of responses.

In this realist review, we will generate context-mechanism-outcome configurations for community engagement in any of the steps of the MPDSR cycle highlighted above.

### Stage 2: Developing candidate Initial Programme Theories

Realist programme theories provide provisional logic models on how community engagement in MPDSR is theorized to work ^56^. We conducted an initial scoping review on community engagement in MPDSR to identify some relevant papers that we could use for generating our initial programme theories. Our initial programme theories (IPTs) are based on the assumption that community members are engaged in the MPDSR process so that they can contribute to achieving the goals of MPDSR for surveillance and response ^3^.

See Table 1 for the initial programme theories (IPTs) on community engagement in the different parts of the MPDSR action cycle.

**Table 1.** Initial Programme Theories for community engagement in MPDSR.

Steps in MPDSR cycle	Goal of CE in MPDSR (in that part of the cycle)
Identification and notification of deaths	Identify and notify all maternal and perinatal deaths occurring in the community and provide the information to health workers.
Review of maternal/perinatal deaths and identification of actions to address modifiable factors identified in the review process	Community members provide information to facilitate classifications for assigning cause of death through verbal autopsy. Community members involved in verbal and social autopsy sessions to discuss maternal/perinatal contributors of specific deaths in the community. Community members participate in death reviews at health facilities and provide information on social factors prior to arriving at a health facility and experiences of care within health facilities to MPDSR committees. Community members involved in verbal and social autopsy sessions to propose community -level actions to address modifiable or avoidable factors identified during the review process.
Response	Community involvement in implementing community-level actions to prevent maternal/perinatal deaths
Monitor and evaluate	Community members involvement in advocacy with duty bearers/health workers and policy makers to support implementation of recommended actions.

From the initial scoping search, we identified 16 papers that described community engagement in the process of identification, notification, review and response of maternal and perinatal deaths. We identified four initial programme theories. These are:

(i) Community engagement supports data collection and facilitates the reporting of maternal and perinatal deaths occurring in the community corresponding with the identification, notification and review steps of the MPDSR cycle ^57– 59^.
(ii) Community engagement supports quality of care by providing information to MPDSR committees or being included in MPDSR committees to conduct death reviews. Health professionals can engage community members in MPDSR through social and verbal autopsy for example as a means of improving care seeking and getting feedback from community members on quality of care issues such as disrespectful maternity care ^4, 16, 36^.
(iii) Community members can participate by making recommendations and implementing local level solutions to address some of the material and social barriers identified as contributors to maternal or perinatal deaths; e.g. supporting transport arrangements for pregnant women to facilitate timely delivery ^16, 43^.
(iv) Community members can be involved in advocacy with duty bearers/health providers and policy makers to support implementation of recommendations ^16, 43^.

From the initial scoping search, we identified several contexts in which community engagement in MPDSR is implemented. For instance, in some contexts, community engagement is part of a national programme with national guidelines for implementation while in other contexts, the process is implemented in a selected health facility. Similarly, the policy contexts vary e.g., where there are legal processes to anchor the no blame policy while others have no legal framework.

**Figure 2.**
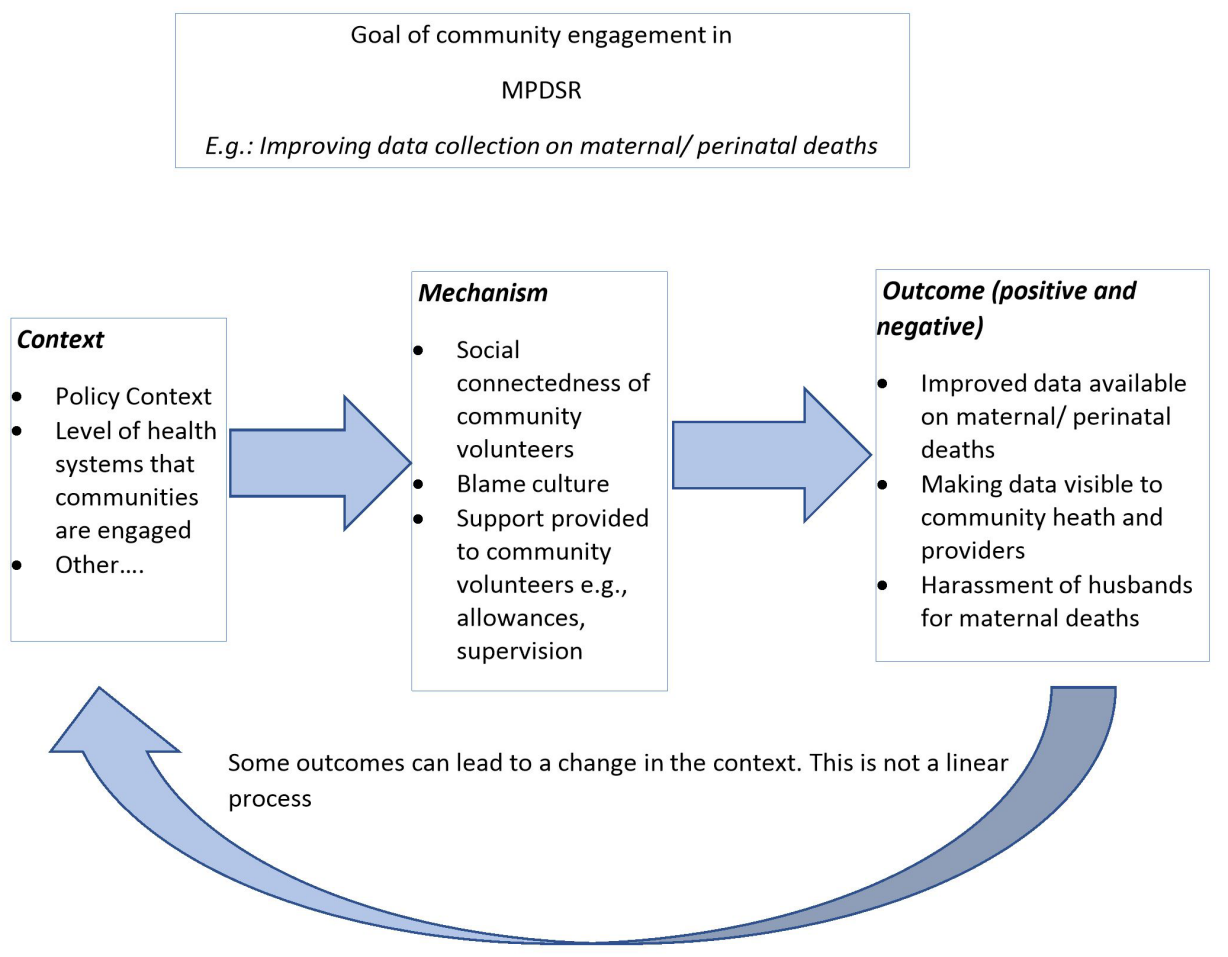
Example of CMO configurations adapted from: 60.

We will explore the different contexts in which community members are engaged and identify the mechanisms that are triggered and the outcomes they produce. See Figure 2 for an example of potential CMOCs.

### Stage 3: Setting up steering advisory group

We have set up an advisory group to guide the process of identifying the initial programme theories and subsequent refinement of the theories. We are working with the WHO Technical Working Group (TWG) for community engagement and blame culture as the advisory group for this realist review. The WHO TWG is made up of academics, health professionals and NGO representatives working in MPDSR globally.

The advisory group reviewed the search terms proposed for this realist review to ensure that the process is comprehensive. We presented the initial programme theories on community engagement in MPDSR to the advisory group for their review and comments. Based on the feedback from the advisory group, we have identified programme theories that line up with the overall goal of MPDSR and the forms of community engagement in different parts of the MPDSR cycle as shown above.

### Stage 4: Searching for evidence

During the scoping for literature stage 1 above, we realized that there are very few articles that report on MPDSR interventions that complete the MPDSR cycle. As such, for this review, we will include publications or reports that provide sufficient detail on any aspect of maternal or perinatal death reviews, verbal, or social autopsy even if the articles do not describe the full MPDSR cycle. Based on our discussions with the steering advisory group, articles that do not report on the entire MPDSR action cycle would still be useful for explaining how community engagement works in specific steps of the cycle. We will also include papers that describe broad aspects of MPDSR such as community involvement in surveillance of deaths to generate theory on how community members can support surveillance efforts in MPDSR.

We will conduct a search for relevant papers and articles in both published and grey literature. The search terms will then be refined with the assistance of a librarian from the London School of Hygiene and Tropical Medicine (LSHTM). They will be divided into three search concepts: ‘community engagement,’ ‘maternal and perinatal death,’ and ‘surveillance and response’. The search will be conducted on six databases to identify peer-reviewed articles: Medline, Embase, Global Health, CINAHL Plus, Scopus and Web of Science.

Keywords or free text terms will be developed for each of the search concepts and thesaurus searching will be used to identify synonyms and other relevant terms. Several search techniques will be employed to make the search more comprehensive and focused. Truncation will be used with keywords to specify different ending to words; wildcards will be used to make allowances for differences in spelling; whilst proximity search will be used to allow combination of words in a different order.

For Ovid interface, medical subject headings (MeSH) will be exploded where applicable. The search in Ovid interface will be conducted in Medline database initially and then replicated in Embase and Global Health databases. Alternatives to keywords and subject headings will be made to suit CINAHL Plus and Web of Science databases. The Boolean operator ‘OR’ will be used to retrieve records/references unique to each search concept and the operator ‘AND’ will be used to combine all three search concepts.

We will conduct iterative searches by hand searching reference lists and bibliographies of relevant articles that may contribute to theory refinement or rebuttal ^56^. We may also conduct cluster searching, where we identify any additional papers that may be related to a specific study that is relevant for inclusion in this realist review ^52^.

We will conduct a grey literature search using the same search concepts on Google Scholar.

In addition, we will consult experts in the field, NGOs implementing projects on MPDSR and members of the WHO’s MPDSR Technical Working Group (TWG) to ensure any other relevant articles (peer-reviewed and grey) are identified.

### Search terms

The search terms in this review are adapted and updated from two reviews: Marston *et al.* for community engagement search terms ^22^; and Kinney *et al.* for MPDSR search terms ^61^. We identified these search terms in light of the Initial Programme Theories (IPTs) to facilitate retrieval of relevant articles.

See Table 2 for a comprehensive list of the search terms.

**Table 2.** Search terms for community engagement in MPDSR.

Community Engagement terms	“Collective or community or community intervention” or “community action” or “community mobilisation” or “capacity building” or collaboration or conscientization or engagement or intervention or outreach or involvement or consultation or “shared leadership” or “community network” or “community participation” or leadership or “health program” or “community initiative” Empower* or “Health Promotion” or “Maximi? ing access” or “Participatory intervention” or “Participatory approach” or “Social mobilization” or “Social movement” or “Social capital” or “Social participation” or “Village health worker” or “Women group” or “community capability” or “collective efficacy” or “patient public involvement” or PPI or “patient public engagement” “Consumer participation” or engagement or involvement or “community representation” or “community accountability” or “community W3 accountability” or representation or “social accountability” or “community advocacy” or “community health worker” or “community representative” or “health facility committee” or “health management committee” or “Stakeholder participation” or “stakeholder engagement” or “health co-production”
Maternal or Perinatal death	“Maternal death” OR “mother death” OR maternity OR fetal OR perinatal OR pregnancy OR "child-birth" OR birth OR "labo?r W/3 mortality" OR death* OR fatality* OR “pregnancy complication” OR “f?etal death” OR “still-birth” OR “still-born” OR “sudden infant death” OR sids OR “cot death” OR “crib death” or “saving mothers lives” OR “making pregnancy safer” OR “making childbirth safer” OR “new-born death” OR “intrapartum death” OR “intrapartum mortality”
Surveillance and Response	"maternal and perinatal death surveillance and response" or MPDSR or “maternal death surveillance and response” or MDSR or audit or surveillance or response or "death audit" or “maternal death review” or perinatal death review” or "death surveillance" or "death review" or "surveillance W3response" or "confidential enquir*" or "confidential inquir*" or "death* meeting" or "death enquir*" or "death inquir*" or "confidential enquir* into Maternal and Child Health" or CEMACH or "Confidential Inquir* into Maternal and Child Health" or CIMACH or "Cent* for Maternal and Child Enquir*" or CMACE or "Cent* for Maternal and Child Inquir*" or CMACI or "Confidential Enquir* into Maternal Death" or CEMD or "Confidential Inquir* into Maternal Death" or CIMD or "Cent* for Maternal Death Enquir*" or CMDE or "Cent* for Maternal Death Inquir*" or CMDI or "verbal autops*" or "social autops*" or "communit* W3 death audit" or "death review" or "death meeting" or "verbal autops*" or "social autopsy"

### Stage 5: Conducting the search, screening, and initial data extraction

***Eligibility criteria.*** The literature search for this realist review is limited to papers published from 2004 to coincide with the publication of the first WHO maternal death review guideline, ‘Beyond the Numbers’ ^62^. The search will cover countries from all income levels and include articles in any language. Where necessary, translations of papers in any other languages other than English will be sought.

This realist review will include published papers and grey literature that can contribute to theory building or testing of community engagement in MPDSR. It will also include any commentaries or opinion pieces that emphasize the need for community engagement. Any article that describes an aspect of community engagement in MPDSR/MDSR or perinatal death reviews (PDRs) will be included. For instance, an article that describes community involvement in collecting information about deaths or doing social/verbal autopsies to improve maternal and new-born health without feeding into an audit or review process will be included. But studies focusing on the effectiveness of maternal and/or perinatal death review or MDSR or MPDSR implementation at the facility level with no community engagement will be excluded.

***Screening.*** All published articles and grey literature identified through the different approaches will be uploaded onto Eppi-Reviewer 4 for screening on title and abstract. Two members of the team (MMb and IO) will double screen the articles on title and abstract. Two other members of the team (LPK and AP) will re-screen 10% of the articles on title and abstract to ensure rigour in the process. This will be followed by a full text screening to identify papers that are theory rich, i.e., they provide sufficient detail to either refine or refute the initial programme theories. We will provide details to show the screening process following the PRISMA diagram.

### Stage 6: Data extraction and analysis

We have developed a data extraction tool that we will adjust iteratively and populate with evidence on context-mechanism and outcome configurations. We will use both inductive and deductive reasoning and our own insights and common sense to understand generative causation ^56^. We will begin with an inductive approach to coding to identify the context-mechanisms and outcomes in the articles. For the abductive analysis, we will draw on different theories that may have relevance for this review for instance we could explore the programme theories for community engagement in quality improvement programmes or health promotion programmes to give pointers for community engagement in MPDSR ^52, 56^.

The first author will extract data from the included articles to identify the context-mechanism: outcome configurations. In our analysis, we will consider how issues of gender and sex influence specific CMOs and reflect these similarities or differences in the refinement process. These configurations will be discussed with co-authors and the advisory group for refinement. We will produce CMOs that report both positive and negative outcomes of community engagement in MPDSR.

### Stage 7: Refining programme theories

After discussions on the emerging IPTs from the data extraction exercise, we will present the IPTs to the advisory group and the WHO TWG’s sub-group on community engagement and blame culture to test the theories. The advisory group is made up of individuals with different capacities and expertise with regard to MPDSR implementation at different levels ranging from the community to global initiatives. We will leverage on this expertise to ensure that the refined theories are robust and relevant to the different contexts where MPDSR is implemented.

### Quality appraisal

Realist reviews synthesize different kinds of evidence: qualitative, quantitative or mixed methods study designs to explain the linkages between context, mechanisms and outcomes ^52, 53^. We will use the CASP checklist for quality assessment of peer reviewed studies and the AACODS checklist for grey literature ^63, 64^.

Studies will be included primarily on the basis of relevance and the extent to which they contribute to the development and testing of theories on community engagement in MPDSR ^52, 56^. We will appraise papers for rigor and relevance, giving scores of ‘high’, ‘moderate’ or ‘low’ with regard to the extent to which an article provides details that are useful for generating theory on community engagement in MPDSR ^65^. We will use the RAMESES standards for reporting realist reviews ^53^.

### Study status

We have conducted an initial search using the search terms described in this paper. Screening of papers and grey literature is on-going.

### Dissemination and next steps

We will disseminate the findings of the realist review to multiple stakeholders. These include the WHO MPDSR TWG and its sub-group on community engagement and blame culture. It is expected that the findings can be used to guide the development of training tools for engaging community members in MPDSR that are relevant to the different contexts where MPDSR is implemented. We expect that the programme theories generated through this review can be relevant to broader issues of community engagement in maternal and newborn health beyond MPDSR.

We will publish the findings and conclusions of the realist review in a peer reviewed journal as well as through conferences. We will leverage on our participation in the WHO global MPDSR Technical Working Group to disseminate the findings to a global audience.

### Limitations

The realist review relies on the richness and adequacy of descriptions in original studies. Where details on community engagement are lacking, this could limit programme theory development. To mitigate this, we will contact authors of relevant papers for additional details that could enrich programme theory development.

### Ethics

As this review is a synthesis of existing literature, we do not require ethics approval. But we will ensure that the review process is transparent by carefully documenting the processes that we will follow and the decisions that we make when refining the programme theories.

## Data Availability

No data are associated with this article.

## Acronyms

BTN - Beyond the Numbers

CASP - Critical Appraisal Skills Programme

CMOC - Context-Mechanism-Outcome Configurations

CE - Community Engagement

CSO - Civil Society Organization

IPT - Initial Programme Theory

LSHTM - London School of Hygiene and Tropical Medicine

MDSR - Maternal Death Surveillance and Response

MPDSR - Maternal and Perinatal Death Surveillance and Response

NGO - Non-Governmental Organization

PDR - Perinatal death review

SDG - Sustainable Development Goals

TWG - Technical Working Group

WHO - World Health Organization
