# Supplementary material for: Community engagement in maternal and perinatal death surveillance and response (MPDSR): Realist review protocol
Source: Wellcome Open Res. 2023 Oct 5;8:117. Originally published 2023 Mar 15. [Version 2] doi: 10.12688/wellcomeopenres.18844.2 (PMC10465996; doi:10.12688/wellcomeopenres.18844.2)
Supplement: Peer review history

# Reviewer report: Reviewer response for version 2

**DOI:** 10.21956/wellcomeopenres.22316.r68110

**Published:** 2023-10-31

**Authors:** Gouranga Dasvarma

**Affiliations:** 1 College of Humanities, Arts and Social Sciences, Flinders University, Adelaide, Australia

I approve the revisions.

Is the study design appropriate for the research question?

Yes

Is the rationale for, and objectives of, the study clearly described?

Yes

Are sufficient details of the methods provided to allow replication by others?

Yes

Are the datasets clearly presented in a useable and accessible format?

Not applicable

Reviewer Expertise:

Mortality and Health; Fertility and family planning; population and development; population and enviornment

I confirm that I have read this submission and believe that I have an appropriate level of expertise to confirm that it is of an acceptable scientific standard.

# Reviewer report: Reviewer response for version 2

**DOI:** 10.21956/wellcomeopenres.22316.r68111

**Published:** 2023-10-20

**Authors:** David Roger Walugembe

**Affiliations:** 1 School of Health Studies and Faculty of Information and Media Studies, The University of British Columbia, Vancouver, British Columbia, Canada

I have read the revised version and can confirm that the changes made by the authors are appropriate and sufficient for me to amend my previous status of Approved with reservations. As such, I would like to observe that my current status is  "Approved". 

 One additional comment though is that if possible, the close quotation marks at the end of the paragraph below can be deleted. That is the only edit I think I can make for now. Else, my decision is "Approved" and thank you for the opportunity to review this manuscript. 

 A mechanism is the generative force that leads to outcomes and often denotes the resources and the reasoning that lead to either a positive or negative outcome 51, 55. For this realist review, examples of mechanisms could be fear of litigation by health professionals. This would arise in contexts where community members can sanction health professionals and take legal action. However, because there is no legal framework on blame culture in MPDSR, health professionals may be unwilling to allow community members to participate in MPDSR processes due to fear of litigation. In this case the fear of litigation is the mechanism that produces a negative outcome. **”**

Is the study design appropriate for the research question?

Yes

Is the rationale for, and objectives of, the study clearly described?

Yes

Are sufficient details of the methods provided to allow replication by others?

Yes

Are the datasets clearly presented in a useable and accessible format?

Not applicable

Reviewer Expertise:

NA

I confirm that I have read this submission and believe that I have an appropriate level of expertise to confirm that it is of an acceptable scientific standard.

# Reviewer report: Reviewer response for version 1

**DOI:** 10.21956/wellcomeopenres.20895.r64519

**Published:** 2023-08-30

**Authors:** Henry Mark Lugobe

**Affiliations:** 1 Mbarara University of Science and Technology, Mbarara, Western Region, Uganda

The authors seek to address an issue of public health importance. Try to understand community engagement in maternal and perinatal death surveillance and response. The have presented the rationale of the study and the methods well.

 Here are a few comments.

 There are some topographical errors that the authors can attend to.

 Under the definition of community, will the inclusion or exclusion of stakeholders such as policy makers (the Political leaders) and other leaders like traditional leaders or Religious leaders be explored?

 Does the MPDSR activity with or without community engagement contribute to the utilization or under utilization of health facilities? and have Any outcomes specific to health workers? Any outcome directed towards better care or reduction in maternal deaths? Would the review be in position to explore some of these as well?

Is the study design appropriate for the research question?

Yes

Is the rationale for, and objectives of, the study clearly described?

Yes

Are sufficient details of the methods provided to allow replication by others?

Yes

Are the datasets clearly presented in a useable and accessible format?

Yes

Reviewer Expertise:

High risk obstetrics, Quality improvement processes for maternal and perinatal death audits, Hypertensive disorders of pregnancy

I confirm that I have read this submission and believe that I have an appropriate level of expertise to confirm that it is of an acceptable scientific standard.

# Reviewer report: Reviewer response for version 1

**DOI:** 10.21956/wellcomeopenres.20895.r64517

**Published:** 2023-08-29

**Authors:** Gouranga Dasvarma

**Affiliations:** 1 College of Humanities, Arts and Social Sciences, Flinders University, Adelaide, Australia

This manuscript contains a well-written proposal for a protocol on community engagement for surveillance of maternal and perinatal deaths. In countries with insufficient detail in the still incomplete record of high levels of maternal and perinatal deaths, the engagement of family members and the community is very important in efforts to reduce maternal and perinatal mortality. The methodology for this realist review is adequately described and, when finalised the protocol for community engagement can be adapted to different situations with appropriate adjustments.

 I have made specific comments/questions in the body of the manuscript which are reproduced below:

- Abstract. Background. Line 2. which deaths remain high?
- Abstract. Background. Line 3. Which SDG?
- Page 3. Community engagement. End of paragraph 1. Does it not give voice to community members in the planning of health service provision?
- Page 6. Heading. Stage 2: Developing candidate initial Programme Theories.
- Shouldn't there be "for" inserted between the words candidate and Initial?
- Page 6. Re four initial programmes. the first programme-
  I suggest taking note of an innovative technique of reconstructing the circumstances of infant deaths- called by its authors The Rashomon Technique. Based on the Japanese film Rashomon, where different witnesses use their own perspective to give different accounts of a rape and murder, the authors of this research in a province in eastern Indonesia try to reconstruct the circumstances surrounding the very high rates of infant deaths occurring in that province.

Please see " *They Simply Die: Searching for the Causes of Infant Mortality in Lombok*" By Rusman, Roosmalawati; Djohan, Eniarti; Hull, Terence H. Jakarta. PPT-LIPI. 1999. xix. 100 pages. 23 cm. ISBN: 979-8553-43-8.

- Page 7. Searching for evidence. Same as the previous comment.
- Other suggested articles for review:
  - Paudel, Mohan; Sara Javanparast; Gouranga Dasvarma and Lareen Newman (2019). “A critical account of the policy context shaping perinatal survival in Nepal: policy tension of socio-cultural versus a medical approach”. *BMC Health Services Research*. (2019) 19:166. https://doi.org/10.1186/s12913-019-3979-3.
  - Paudel, Mohan; Sara Javanparast; Gouranga Dasvarma and Lareen Newman (2018). “A qualitative study about the gendered experiences of motherhood and perinatal mortality in mountain villages of Nepal: implications for improving perinatal survival”. *BMC Pregnancy and Childbirth*. (2018) 18:163. https://doi.org/10.1186/s12884-018-1776-3
  - Paudel, Mohan; Sara Javanparast; Lareen Newman and Gouranga Dasvarma (2018) “Health system barriers influencing perinatal survival in mountain villages of Nepal: implications for future policies and practices”. *Journal of Health, Population and Nutrition* (2018) 37:16.
  - Paudel M, Javanparast S, Dasvarma G, Newman L (2018). “Religio-cultural factors contributing to perinatal mortality and morbidity in mountain villages of Nepal: Implications for future healthcare provision”. PLoS ONE 13(3): e0194328. https://doi.org/10.1371/journal.pone.0194328.

Is the study design appropriate for the research question?

Yes

Is the rationale for, and objectives of, the study clearly described?

Yes

Are sufficient details of the methods provided to allow replication by others?

Yes

Are the datasets clearly presented in a useable and accessible format?

Not applicable

Reviewer Expertise:

Mortality and Health; Fertility and family planning; population and development; population and enviornment

I confirm that I have read this submission and believe that I have an appropriate level of expertise to confirm that it is of an acceptable scientific standard, however I have significant reservations, as outlined above.

## References

[1] : They Simply Die: Searching for the Causes of Infant Mortality in Lombok. *Jakarta. PPT-LIPI* .1999;

[2] : A critical account of the policy context shaping perinatal survival in Nepal: policy tension of socio-cultural versus a medical approach. *BMC Health Serv Res* .2019;19(1) : 10.1186/s12913-019-3979-3 166 10.1186/s12913-019-3979-3 30871523PMC6417211

[3] : A qualitative study about the gendered experiences of motherhood and perinatal mortality in mountain villages of Nepal: implications for improving perinatal survival. *BMC Pregnancy Childbirth* .2018;18(1) : 10.1186/s12884-018-1776-3 163 10.1186/s12884-018-1776-3 29764385PMC5952814

[4] : Health system barriers influencing perinatal survival in mountain villages of Nepal: implications for future policies and practices. *J Health Popul Nutr* .2018;37(1) : 10.1186/s41043-018-0148-y 16 10.1186/s41043-018-0148-y 29976245PMC6034263

[5] : Religio-cultural factors contributing to perinatal mortality and morbidity in mountain villages of Nepal: Implications for future healthcare provision. *PLoS One* .2018;13(3) : 10.1371/journal.pone.0194328 e0194328 10.1371/journal.pone.0194328 29544226PMC5854484

# Reviewer report: Reviewer response for version 1

**DOI:** 10.21956/wellcomeopenres.20895.r60135

**Published:** 2023-08-09

**Authors:** Serena Donati

**Affiliations:** 1 National Center for Disease Prevention and Health Promotion, National Institute of Health, Rome, Italy

The issue addressed by the authors is a public health priority at global level. The research question “Which strategies of community engagement in MPDSR produce which outcomes in which contexts?” looks very appropriate to me even though I wonder if the available literature on community engagement strategies will be enough to "explore and explain what, why, how and for whom it will work". The growing need to gather knowledge on how to involve communities in the implementation of MPDSR and finally improve maternal and perinatal care by reducing avoidable infaust outcomes is still a challenge.

 The methodology adopted is well described and robust, the organization of the document, the logical sequence of its sections, the selection of the bibliographic references and the interpretation of the available evidence are excellent and denote knowledge and mastery of the subject.

 Reading is enjoyable and engaging. I, therefore, thank the authors for this valuable protocol and wish them success in developing operational guidelines that will have an impact in promoting MDRS in contexts where maternal and perinatal mortality are still unduly high.

Is the study design appropriate for the research question?

Yes

Is the rationale for, and objectives of, the study clearly described?

Yes

Are sufficient details of the methods provided to allow replication by others?

Yes

Are the datasets clearly presented in a useable and accessible format?

Not applicable

Reviewer Expertise:

Public health research, maternal and perinatal health, maternal and perinatal death surveillance and response

I confirm that I have read this submission and believe that I have an appropriate level of expertise to confirm that it is of an acceptable scientific standard.

# Reviewer report: Reviewer response for version 1

**DOI:** 10.21956/wellcomeopenres.20895.r55581

**Published:** 2023-04-17

**Authors:** David Roger Walugembe

**Affiliations:** 1 School of Health Studies and Faculty of Information and Media Studies, The University of British Columbia, Vancouver, British Columbia, Canada

This is a well written manuscript and timely as the need to understand how best to engage communities in the implementation of MPDSR becomes increasingly critical. I commend the authors for taking the initiative to contribute towards enhanced understanding of how best this can be done.

 A few observations and suggestions

 [Be] needs to be added in the sentence below *“MPDSR can - implemented using different tools or strategies in different contexts. These include maternal and/or perinatal death reviews, community-based reviews and confidential enquiries into maternal deaths* *8* *.”*

 Considering that there are weak surveillance systems at community level, won’t this be another limitation to this realist review? Will the realist review seek to establish whether studies reporting about community engagement have/had such systems to capture data on this aspect?

*The overall magnitude of mortality trends remains unclear because of weak surveillance systems especially at the community* *14* *.*

 Identify instead of identifies may be ideal in the sentence below

*These include WHO technical guidelines on maternal death surveillance and* *response (MDSR)* *2* *, which* *identifies* *community members as a critical stakeholder in surveillance because they can provide information on the social factors that contribute to maternal deaths.*

 The explanation of a “mechanism” can be improved upon because its currently lost in the phrasing/structure of the sentence. A possible suggestion for phrasing this sentence may go along the lines of: 

*“For this realist review, examples of mechanisms could be fear of litigation by health professionals. This would arise in contexts where community members can sanction health professionals and take legal action. However, because there is no legal framework on blame culture in MPDSR, health professionals may be unwilling to allow community members to participate in MPDSR processes due to fear of litigation. In this case the fear of litigation is the mechanism that produces a negative outcome.”*

*“We propose a realist review approach* *33* *to explore and explain what, why, how and for whom community engagement in MPDSR works (or does not work) to support implementation of MPDSR throughout the action cycle.”*

 Since a realist review approach was proposed to achieve the goals below, would any of the suggested rephrasing for the aim this realist review appeal to the authors or it would distort the meaning/purpose *?*

- Which MPDSR Community engagement strategies work in which context and what/which outcomes do they produce? or
- What MPDSR community engagement strategies work in which/what contexts, what outcomes do they produce and for who?

Could the authors provide references/citations for the following elements to facilitate possible replication of these steps by future scholars and users of the content of this article;

- *For Ovid interface, medical subject headings (MeSH) will be exploded where applicable (ref).*
- *All published articles and grey literature identified through the different approaches will be uploaded onto Eppi-Reviewer 4 (ref) for screening on title and abstract*

Would the authors consider using “however” instead of “but” in the sentence below or keep it as one sentence with a comma after approval?

*As this review is a synthesis of existing literature, we do not require ethics approval. But we will ensure that the review process is transparent by carefully documenting the processes that we will follow and the decisions that we make when refining the programme theories.*

 Lastly, given the crosscutting importance of community engagement in MPDSR implementation, I tend to think that dissemination will benefit all the WHO Technical sub working groups including blame culture, capacity building   and monitoring and reporting among others.

Is the study design appropriate for the research question?

Yes

Is the rationale for, and objectives of, the study clearly described?

Yes

Are sufficient details of the methods provided to allow replication by others?

Yes

Are the datasets clearly presented in a useable and accessible format?

Not applicable

Reviewer Expertise:

Implementation science, integrated knowledge translation, health research policy and systems, maternal and child health, stakeholder engagement, sustainability and health equity

I confirm that I have read this submission and believe that I have an appropriate level of expertise to confirm that it is of an acceptable scientific standard, however I have significant reservations, as outlined above.
